# Driver's turning intent recognition model based on brain activation and contextual information

**DOI:** 10.3389/fnrgo.2022.956863

**Published:** 2022-08-16

**Authors:** Alexander Trende, Anirudh Unni, Mischa Jablonski, Bianca Biebl, Andreas Lüdtke, Martin Fränzle, Jochem W. Rieger

**Affiliations:** ^1^German Aerospace Center, Institute of Systems Engineering for Future Mobility, Oldenburg, Germany; ^2^Applied Neurocognitive Psychology Lab, Department of Psychology, University of Oldenburg, Oldenburg, Germany; ^3^School of Engineering and Design, Technical University of Munich, Garching, Germany; ^4^Foundations and Applications of Systems of Cyber-Physical Systems, Department of Computing Science, University of Oldenburg, Oldenburg, Germany

**Keywords:** fNIRS, machine learning, neuroergonomics, driving simulator, intention classification, automotive

## Abstract

Traffic situations like turning at intersections are destined for safety-critical situations and accidents. Human errors are one of the main reasons for accidents in these situations. A model that recognizes the driver's turning intent could help to reduce accidents by warning the driver or stopping the vehicle before a dangerous turning maneuver. Most models that aim at predicting the probability of a driver's turning intent use only contextual information, such as gap size or waiting time. The objective of this study is to investigate whether the combination of context information and brain activation measurements enhances the recognition of turning intent. We conducted a driving simulator study while simultaneously measuring brain activation using high-density fNIRS. A neural network model for turning intent recognition was trained on the fNIRS and contextual data. The input variables were analyzed using SHAP (SHapley Additive exPlanations) feature importance analysis to show the positive effect of the inclusion of brain activation data. Both the model's evaluation and the feature importance analysis suggest that the combination of context information and brain activation leads to an improved turning intent recognition. The fNIRS results showed increased brain activation differences during the “turn” decision-making phase before turning execution in parts of the left motor cortices, such as the primary motor cortex (PMC; putative BA 4), premotor area (PMA; putative BA 6), and supplementary motor area (SMA; putative BA 8). Furthermore, we also observed increased activation differences in the left prefrontal areas, potentially in the left middle frontal gyrus (putative BA 9), which has been associated with the control of executive functions, such as decision-making and action planning. We hypothesize that brain activation measurements could be a more direct indicator with potentially high specificity for the turning behavior and thus help to increase the recognition model's performance.

## Introduction

Driving is a complex and potentially dangerous task. Especially maneuvers like merging and turning are predestined for safety-critical situations and accidents (Yan et al., 2007). Human errors are one of the most common reasons for vehicle collisions (Singh, 2015). Turning through oncoming traffic at unsignalized intersections contributes to 7.4% of all non-severe vehicle crashes in the United States of America (Harding et al., 2014). Human error, in particular, the incorrect estimation of the gap size with respect to the oncoming vehicle, is one of the main reasons for accidents in these situations (Plavsic, 2010). Detecting the driver's intention before such a safety-critical maneuver could give an advanced driver assistance system enough time to initiate a risk-mitigating action. Such intention recognition models could potentially be used to warn the driver before or during a dangerous maneuver, and thus reduce the risk or criticality of such a situation. Furthermore, with the advent of Vehicle-to-Vehicle or Vehicle-to-Infrastructure technology (Harding et al., 2014), the intent recognition results could be used to activate warning or intervention strategies in the oncoming vehicle. An example could be to warn the oncoming vehicle's operator *via* a graphical or auditory interface or directly slowing down the oncoming vehicle's speed and thereby increase the gap size subsequently. Introducing other sensors in addition to vehicle sensors could help to account for variability in user-specific factors that influence turning intention.

Several researchers investigated turning at intersections, to understand the driver's intention and decision-making process through observation of contextual information like gap size, waiting time, etc. Based on video recordings in the USA, Ragland et al. (2005) identified gap acceptance statistics for turning maneuvers at intersections. The researchers then used a logit model to predict the gap acceptance probability based on the given data. But gap size is not the only factor that affects decision-making at intersections. Hamed et al. (1997) developed a multiple regression model to predict the mean critical gap for turning maneuvers. Based on observations of and interviews with drivers across 15 urban intersections, the researchers found that, in addition to the waiting time at intersections (Fricker et al., 1991; Zohdy et al., 2010), the time of the day and the purpose of the trip can also influence the average selected gap for turning maneuvers. Zohdy et al. (2010) investigated different factors, such as the critical gap size, waiting time at the intersections, weather, and the overall travel time, as independent variables for the decision-making process in a left-turn scenario. The authors concluded that drivers became more aggressive as they waited longer at the intersection, leading to filtering through smaller gap sizes. A driving simulator study performed by Yan et al. (2007) showed that other factors, such as gender and age also have an impact on the gap acceptance at unsignalized left-turns. In the last two decades, multiple researchers investigated machine learning models to classify or predict driver's intent. Klingelschmitt et al. (2014) used a Bayesian network to estimate four different intentions while using vehicle behavior and contextual data about the current traffic situation. The model can predict whether a vehicle will most likely go straight, stop at the traffic light, turn, or follow the leading vehicle. The authors found that the anticipated velocity at the stop line is one of the best indicators for intention prediction. Both studies use dynamic vehicle information, like vehicle speed or position, for the intention classification. However, these predictors only vary if the vehicle is not already waiting at the intersection and thus cannot include waiting time. Phillips et al. (2017) used a long short-term memory network to predict whether a driver will turn left, turn right, or drive straight at an intersection. The model uses different driving dynamic information, like speed or acceleration, but also contextual information about the layout of the intersection. The model is able to predict the correct driver intention with an average accuracy of 85%. Zhang and Fu (2020) used a hybrid approach to predict turning intention at an intersection. The researcher uses an ARIMA time-series prediction to estimate vehicle dynamic parameters, like acceleration and speed and afterward predict whether the vehicle will turn left, right, or go straight with a bidirectional long short-term memory network. The optimal recognition rate of 94.2% was achieved 1 s before the maneuver. Trende et al. (2021) used a Bayesian network to classify the turning intention at unsignalized intersections in a driving simulator study. Their turning intent was classified while the vehicle was waiting at the intersection. Besides contextual information, such as gap size and waiting time, that study also included user information, such as gender and age, and results of a driving style questionnaire. Importantly, a feature importance analysis showed that the contextual information contributed the most to the reliable recognition of the turning intention. Therefore, we focus here on gap size and waiting time.

In addition to contextual information, neuroimaging techniques, such as functional near-infrared spectroscopy (fNIRS), have the potential to provide an alternative information channel to design and develop portable brain-based driving assistance decision support systems capable of predicting the driver's intent. These techniques make it possible to investigate processes in a driver's brain while performing decision-making tasks, such as turning at intersections.

In fact, previous neuroimaging studies have characterized brain areas involved in decision-making in the context of game-theoretic frameworks. Moreover, neurophysiological research has revealed key neural correlates, such as the dorsolateral prefrontal cortex (dlPFC), ventromedial prefrontal cortex (vmPFC), frontal cingulate, anterior orbito-, and mediofrontal cortices for action-based value signals in decision-making tasks (Sanfey, 2007; Lee, 2008; Rangel et al., 2008; Gläscher et al., 2009; Ruff and Fehr, 2014). However, very few studies in this field have attempted to predict and model human intent from brain activation in realistic situations. Hollmann et al. (2011) employed real-time fMRI to predict online social decisions in the ultimatum game from brain activation and to reveal brain areas that signal whether offers were subjectively perceived as unfair. These approaches have been extended from relatively simple operant conditioning in laboratory environments (Schultz, 2002) to decision-making in a social context (Sanfey et al., 2003; Sanfey, 2007).

Zhu et al. (2019) performed a driving simulator study with fNIRS to compare various classifiers for braking intention prediction. The researchers compared seven different machine-learning algorithms. The authors concluded that a combined model featuring several classifiers achieves the best test accuracy with around 90%. Deep learning has been shown to outperform classic machine-learning models in several tasks. One of the main advantages of such deep-learning models is their flexibility that allows these models to learn important representations and reveal relations in neuroimaging data (Plis et al., 2014). Several researchers have used neural networks and deep learning for classification tasks based on fNIRS brain activation data (Hennrich et al., 2015; Naseer et al., 2015; Evgin et al., 2019; Huve et al., 2019; Tanveer et al., 2019). Lin et al. (2018) used a Linear Discriminant Analysis to offline classify turning maneuvers (left, right, and braking) based on an electroencephalograph. The researchers compared different time window lengths. The optimal model achieved an average single trial classification accuracy of 70.25%. However, to the best of our knowledge, no study combined contextual information and neuroimaging data into an integrated model to predict behavior.

The objective of this study is to investigate whether the integration of whole-head fNIRS brain activation measurements and context information is beneficial for a turning intention model, and which brain areas are predictive of turning intent. Contextual data refers to data about the driver's vehicle, other vehicles, and the overall traffic situation during this turning maneuver. We conducted a driving simulator study while simultaneously measuring brain activation using high-density fNIRS. The participants drove multiple left-turn maneuvers at unsignalized intersections during the study. We focus on the situation where the driver has halted at an intersection and waits to perform a left turn through oncoming traffic. A neural network model for turning intention recognition was trained on the experimental data and afterward evaluated in a quantitative and qualitative way. The input variables were analyzed using SHAP feature importance analysis (Lundberg and Lee, 2017) to demonstrate how both context and brain-imaging data contribute and improve the model's performance.

## Materials and methods

The experimental setup and study have been described in greater detail in Unni et al. (2022) and will be briefly summarized in the following sections. It should be noted that Unni et al. (2022) however focused on different aspects of the dataset.

### Participants

Thirteen participants (seven women, aged 21–29 years (Mean ± SD = 23.8 ± 2.61), driving experience: 5.8 ± 2.5 years) took part in the driving simulator study. All participants held a German driving license and received financial reimbursement of 10 € per hour for their participation. The Ethics Committee of Carl von Ossietzky University, Oldenburg approved the experimental procedure.

### Experimental set-up

**Figure 1:**
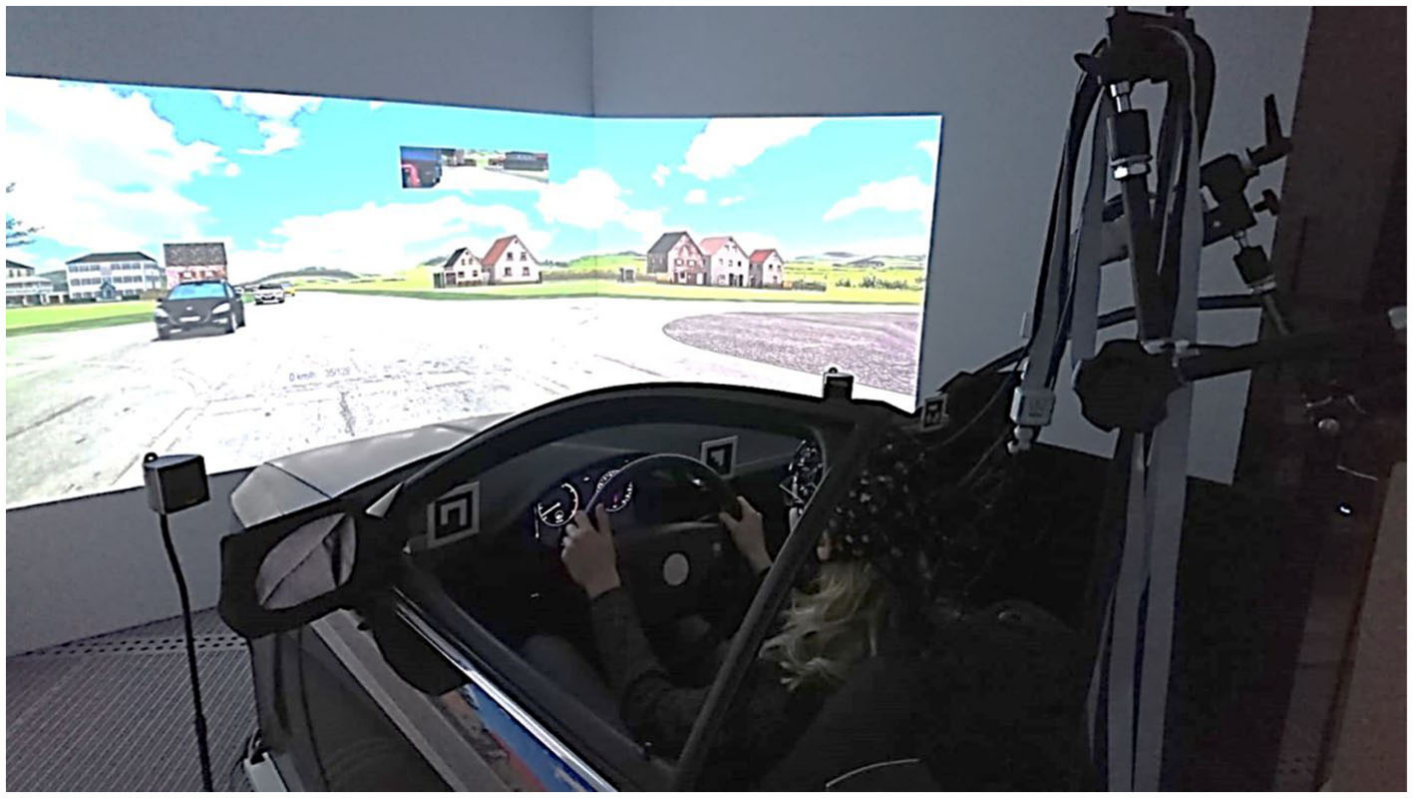
Virtual reality lab driving simulator with the whole-head fNIRS system during the experiment.

The full-scale fixed-based driving simulator used for the experiment has a 150° field of view contained a vehicle mockup and the participants used a standard interface consisting of throttle, brake pedal, and steering wheel (Figure 1).

We used a high-density, whole-head fNIRS system (NIRScout Extended system from NIRx Medical Technologies) to measure the participant's brain activation during the experiment. The system uses near-infrared emitters with 760 and 850 nm wavelengths and outputs relative concentration changes of oxyhemoglobin (HbO) and deoxyhemoglobin (HbR). Thirty-two emitters and detectors to obtain close to whole-head coverage were used. Overall, 107 fNIRS channels (emitter-detector combinations) were used to acquire fNIRS data at a sampling frequency of close to 2 Hz. Both the fNIRS data and the driving simulator data were trigger-synchronized during the driving task. A detailed explanation of the fNIRS setup can be found in Unni et al. (2022).

### Experimental design

**Figure 2:**
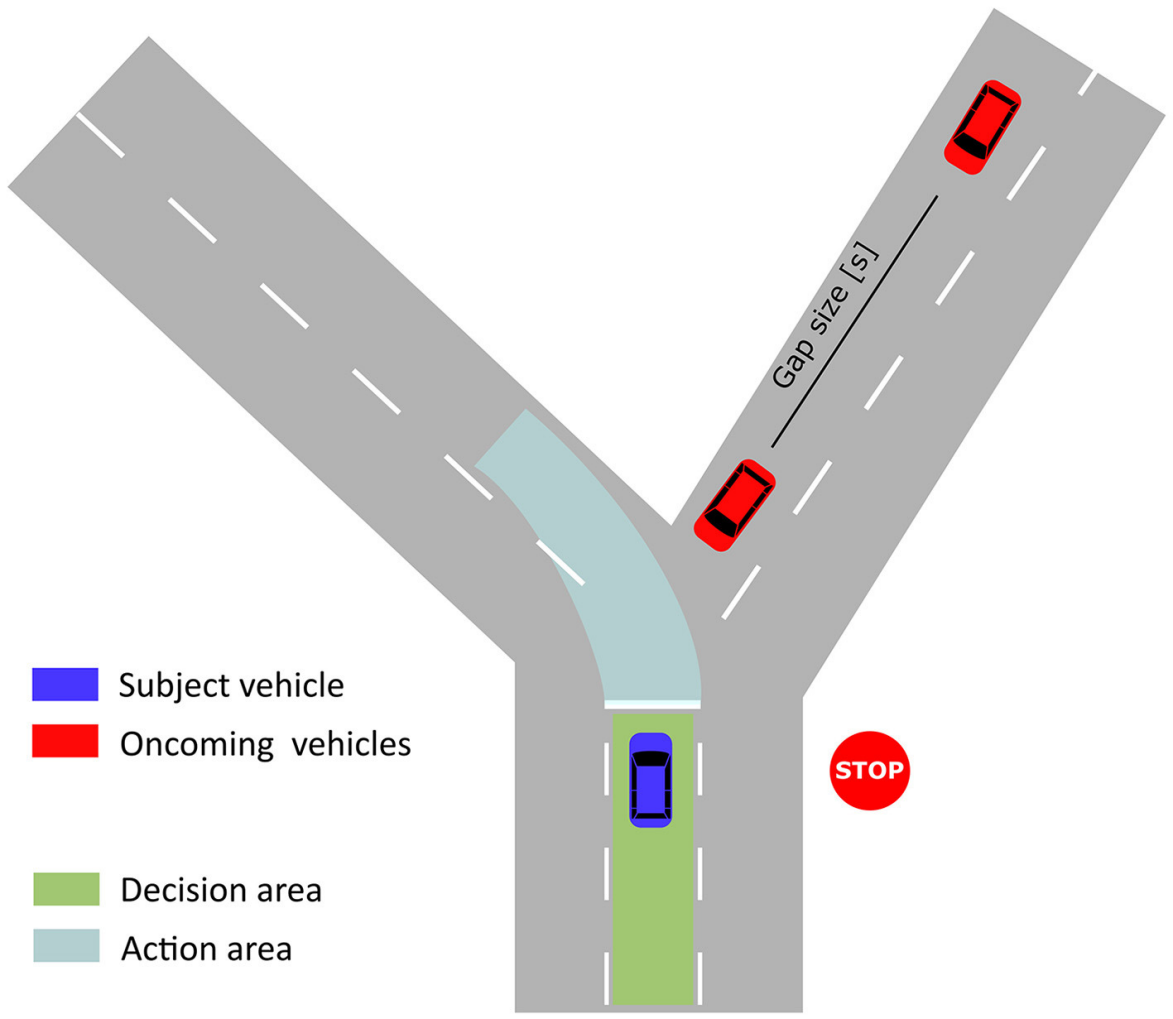
Sketch of intersection. The gap size between two oncoming vehicles is defined as the time that passes after the first oncoming vehicle has crossed the intersection until the second vehicle has crossed the intersection.

The driving scenario consisted of multiple left-turn maneuvers in an urban environment with oncoming vehicles. The oncoming traffic drove at a speed of close to 50 km/h, which is the most common speed limit in urban areas in Germany. The participants had to stop at the intersection due to a stop sign and then had to wait for an appropriate gap in the oncoming traffic. A positive turning intention means that the participant accepts a gap and initiates the turning maneuver. As shown in Figure 2, the lanes following the intersection were bent, which made the estimation of the gap size easier for the participants.

The traffic situations were designed according to the findings of Ragland et al. (2006). We presented gap sizes between 1 and 6 s (14–84 m) during the experiment, whereas the first oncoming cars have smaller gap sizes. This allows finding the minimal acceptable gap size for each participant (Yan et al., 2007). Approximately, 8–10 cars appeared while the participants waited at the intersection and no cars appeared afterward. Each participant drove a training session before the experiment, which took around 10 min. This allowed the participants to get used to the driving simulator and the virtual reality environment. Overall, the participants drove through 100 intersections distributed over 10 blocks with 10 intersections. In total the experimental session took around 70 min to complete. By presenting a time limit to each block, the participants were encouraged to take one of the gaps between the first 10 cars instead of waiting for all of them to pass. The participants received a monetary reward for each block they finished within the time limit. Only 0.16% of all turning maneuvers across all participants were performed after all cars have passed. For each turning decision, the waiting time in a number of cars passed and the chosen gap size were extracted. These two variables are referred to as contextual information in the following sections of the article.

**Figure 3:**
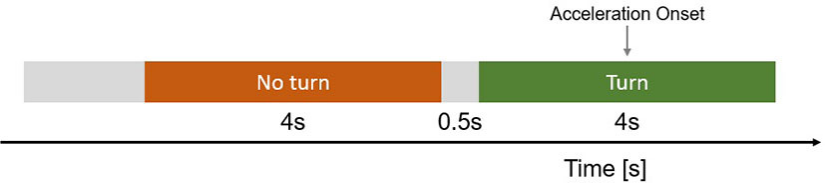
Schema of the data separation procedure. The “turn” time interval was extracted 2 s before the actual initiation of the turning maneuver and thus the depressing of the acceleration pedal. The “no turn” phase was extracted 0.5 s before the “turn” phase to minimize the overlap between the two phases.

The data were labeled based on the two phases: “turn” and “no turn.” Both phases consist of a time window of a 4-s interval (Figure 3). We defined the “turn” phase as the interval focusing on the decision to turn in front of an oncoming vehicle along with the planning to execute the decision. For the fNIRS analysis, we chose the interval 2 s before pressing the accelerator and 2 s after the beginning of the turning maneuver to account for the hemodynamic delay in the BOLD response. This 4 s delay excludes brain activation changes related to motor control during the execution of the turning maneuver. The “no turn” phase is defined as the 4 s time interval before the “turn” phase, in which the participant is waiting for a sufficient gap size at the intersection. A gap between the end of “no turn” and the beginning of “turn” of 0.5 s was chosen to reduce the overlap between the two phases.

### fNIRS data pre-processing

To reduce the influence of systemic physiological artifacts on the fNIRS data, such as cardiac artifacts, respiration rate, or Mayer waves caused by blood pressure oscillations, we pre-processed the fNIRS data using the nirsLAB analysis package (Xu et al., 2014). A detailed explanation of the fNIRS data pre-processing can be found in Unni et al. (2022).

After band-pass filtering [0.01–0.1 Hz] of the raw fNIRS data to reduce the effects of the physiological artifacts along with the very low-frequency drift in the fNIRS data, we performed a visual inspection and all channels that were excessively noisy with various spikes were removed from the analysis. On average, 99 fNIRS channels per participant (SD = 8.7) were included. Then, we applied the modified Beer-Lambert's law to convert the raw data from voltage (μV) to relative concentration change (mmol/l) (Sassaroli and Fantini, 2004).

In this article, the fNIRS analyses were based only on HbR signals, as these are considered to be less susceptible to systemic physiological artifacts like cardiac pulsation, respiration, or Mayer wave fluctuations than HbO (Obrig et al., 2000; Zhang et al., 2005, 2009; Huppert et al., 2009; Suzuki, 2017). Moreover, other studies reported that HbR tends to correlate stronger with the blood oxygenated level dependent (BOLD) response than HbO (MacIntosh et al., 2003; Huppert et al., 2006; Schroeter et al., 2006; Foy et al., 2016).

The normalized fNIRS data were separated into train and test data. We first performed a principal component analysis (PCA) on the training set. In this way, the fNIRS training data was transformed into a set of linearly uncorrelated variables called principal components (PCs). By this method, the first PC accounted for the largest variance in the data, and each successive component had the largest possible variance while maintaining orthogonality to the preceding components. To increase the signal-to-noise ratio (SNR) and limit further analyses to the data explaining the most possible variance, all PCs with eigenvalues <0.7 were removed as recommended by Jolliffe (1972) on Kaiser's rule (Kaiser, 1958). This resulted in an average of 13 PCs (SD = 2.4) per participant. The PCA eigenvectors of the training set were used to transform the test dataset into PC space (Unni et al., 2022). We removed the first PC as it tends to capture movement artifacts in the fNIRS time series.

### Modeling approach

For each participant, three different deep neural networks (DNNs) were trained and validated. The DNNs differed by the amount and type of input features used. The DNNs contained two hidden layers with 25 neurons each. All hidden layers used the ReLu activation function (Hahnloser et al., 2000) and 10% dropout (Srivastava et al., 2014). One model was trained using only contextual features, namely the gap size and waiting time at the intersection. The second model was trained on the second to eight principal components of the fNIRS brain activation recordings. The third model used both, contextual and neurophysiological input features, which lead to nine features in total. The output layer of the network classifies the turning intention with a sigmoid function into the two classes “turn” and “no turn.” The networks were trained with the ADAM optimizer (Kingma and Ba, 2014) for 500 epochs each. Binary cross-entropy was used as a loss function. Five-fold cross-validation was used for model validation. A total of 1,600 instances of input were available per subject. This leads to a training data set the size of 1,280 for each subject given the 80/20 split of the cross-validation. All input variables, except for the discrete “cars waited” input variable, were standardized to have a mean of zero and a standard deviation of one. Standardization of the brain activity data was performed in 4-s intervals. The models were evaluated based on their accuracy. The accuracy is defined as:


$$\begin{array}{l} Accuracy=\frac{TP+TN}{TP+TN+FP+FN}\; \end{array}$$

where TP, TN, FP, and FN are true positives, true negatives, false positives, and false negatives, respectively. Accuracies were calculated for each participant, fold of the cross-validation, and model. Furthermore, a feature importance analysis was performed on the combined model to evaluate which variables contributed most to the model output. SHAP (Lundberg and Lee, 2017) was used for this purpose. SHAP values can show the impact of a feature and its corresponding value on the model's output for a given sample of the data set. SHAP is based on Shapley values used in coalitional game theory. The Shapley value for a feature *j* can be calculated *via* the following equation (Štrumbelj and Kononenko, 2014):


$$\begin{array}{l} \hat{\phi_{j}}=\frac{1}{M}\;\overset{M}{\underset{m=1}{\sum }}\left(\hat{f}\left(x_{+j}\right)\;-\;\hat{f}\left(x_{-j}\right)\right) \end{array}$$

Here the average difference between the model's prediction $\hat{f}\left(x_{+j}\right)$ is compared to the model's prediction $\hat{f}\left(x_{-j}\right)$. *x*_+*j*_ corresponds to the model's input with random feature values for all features except feature *j*. In *x*_−*j*_ the feature value of feature *j* is also random. This difference is computed *M* times and average afterward. Often the global importance *I*_*j*_ of a feature *j* will be calculated:


$$\begin{array}{l} I_{j}=\frac{1}{n}\;\overset{n}{\underset{i=1}{\sum }}|\phi_{j}^{\left(i\right)}| \end{array}$$

where, $|\phi_{j}^{\left(i\right)}|\;$the Shapley value for feature *j* and sample *i* of the dataset of size *n* is.

For each fold and participant, the mean SHAP values for all input variables were calculated. Furthermore, the loadings of the principal components from the pre-processed fNIRS data were also analyzed to characterize brain areas that contribute most to the model's output.

### Characterization of brain areas predictive of turning maneuver from fNIRS data

We aimed to characterize the brain areas involved in the decision-making phase of the turn maneuver compared to the no-turn maneuver at the intersection. For this, we performed a channel-wise paired *t*-test from the preprocessed fNIRS data for the two conditions “turn” and “no turn” on a single-subject level. From the t-values, we calculated the channel-wise Cohen's *d* values to indicate effect sizes in sensor space.

## Results

Results from 12 participants are reported in the following sections since one participant was excluded due to simulator sickness during the experiment.

### Model evaluation

**Figure 4:**
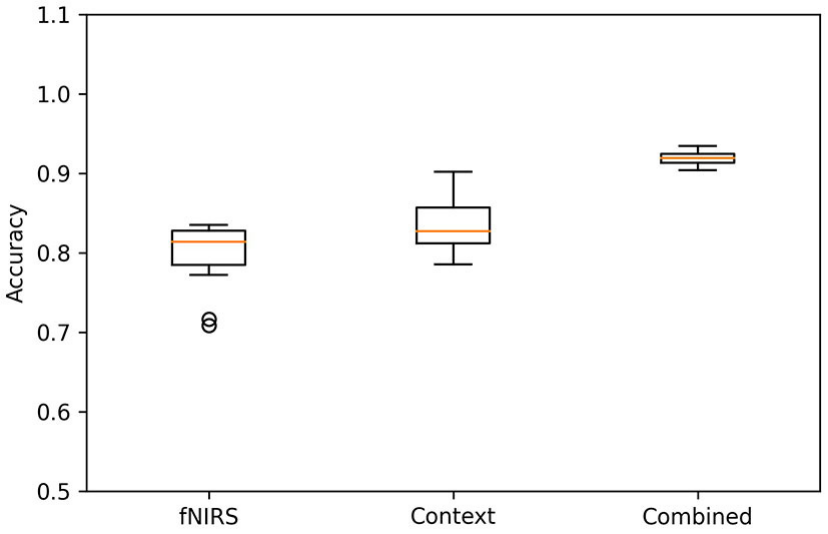
Accuracies for the three models. The figure shows boxplots for all three models, cross-validation folds, and participants.

The first step of the model evaluation was to calculate the accuracy for all three. The metric was calculated for each participant and fold of the cross-validation. The results are presented as boxplots in Figure 4. The fNIRS-alone model achieves a median accuracy of 83.1%. The context-alone model has a median accuracy of 83.8% and the accuracy of the combined model is 91.9%. The corresponding median train accuracy for the three models are 88.7% (fNIRS), 84.0% (context), and 91.9% (combined).

**Figure 5:**
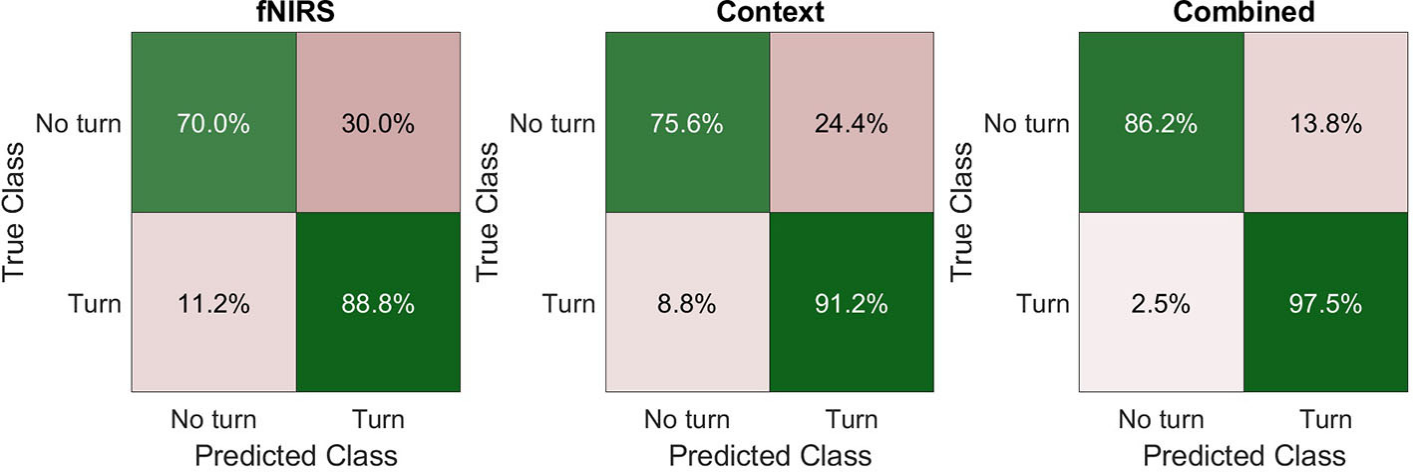
Average confusion matrices for all three models.

**Figure 6:**
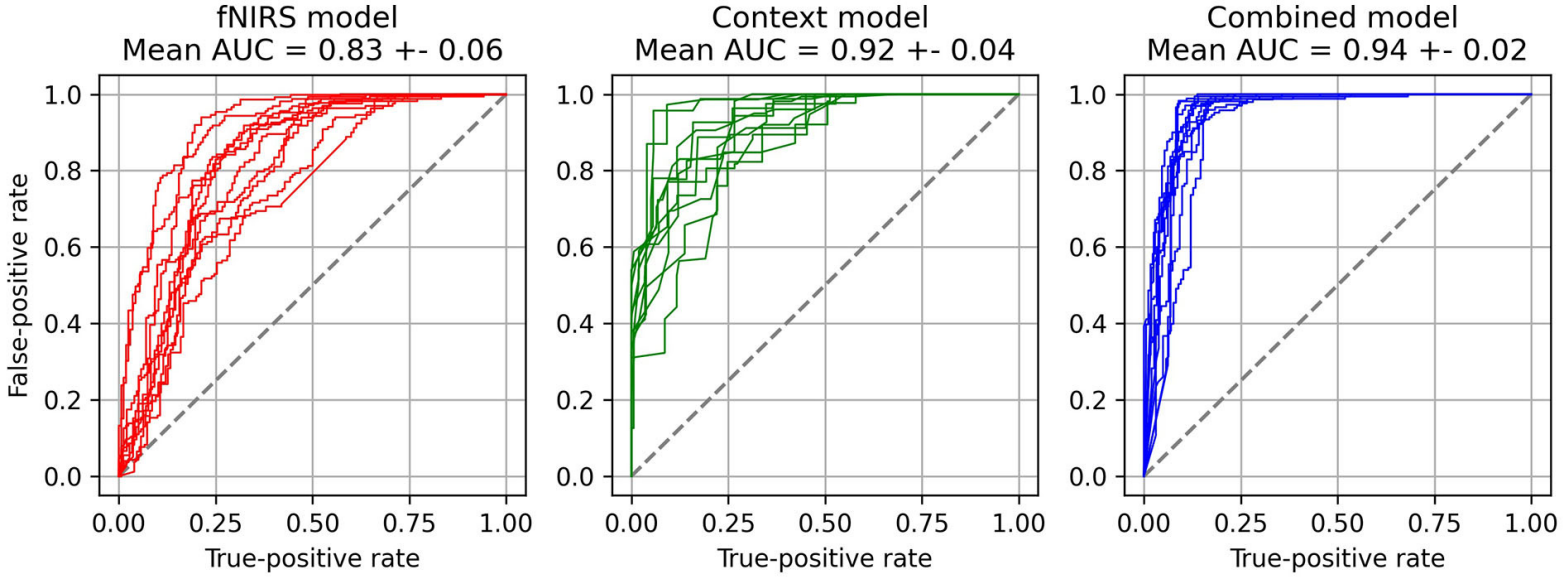
ROC curves for each of the three models and each of the subjects.

Figure 5 shows the average confusion matrices for the three models. The confusion matrix for each subject and fold was calculated and the mean confusion matrix of each model was calculated afterward. Overall, the models were classified as turning best, with at least 88.8% correct classifications. Both the fNIRS and the context model have more than 24% wrongly classified “no turn” instances. In terms of safety, the wrongly classified “turn” instances (false negatives, lower left in the matrices) are the most important ones, as they are the most safety-critical. Both fNIRS and context model misclassify around 10% of these events and predict “no turn” although the participants decided to turn. Importantly, the combined model can significantly reduce this type of misclassification to only 2.5%. Figure 6 shows the receiver operating characteristic (ROC) curves for the three models and one curve per subject. The corresponding average area-under-curve (AUC) and the standard deviation are shown in the title. The combined model has the highest AUC with a value of 0.94 ± 0.02. The context model has a slightly lower AUC of 0.92 ± 0.04 but has larger variability across participants. The ROC curves of the context model for some participants have a comparable AUC to the combined model. However, some curves have a considerably lower AUC. The fNIRS model has an average AUC of 0.83 ± 0.06 and has thus the highest variability of all three models.

### Feature importance analysis

**Figure 7:**
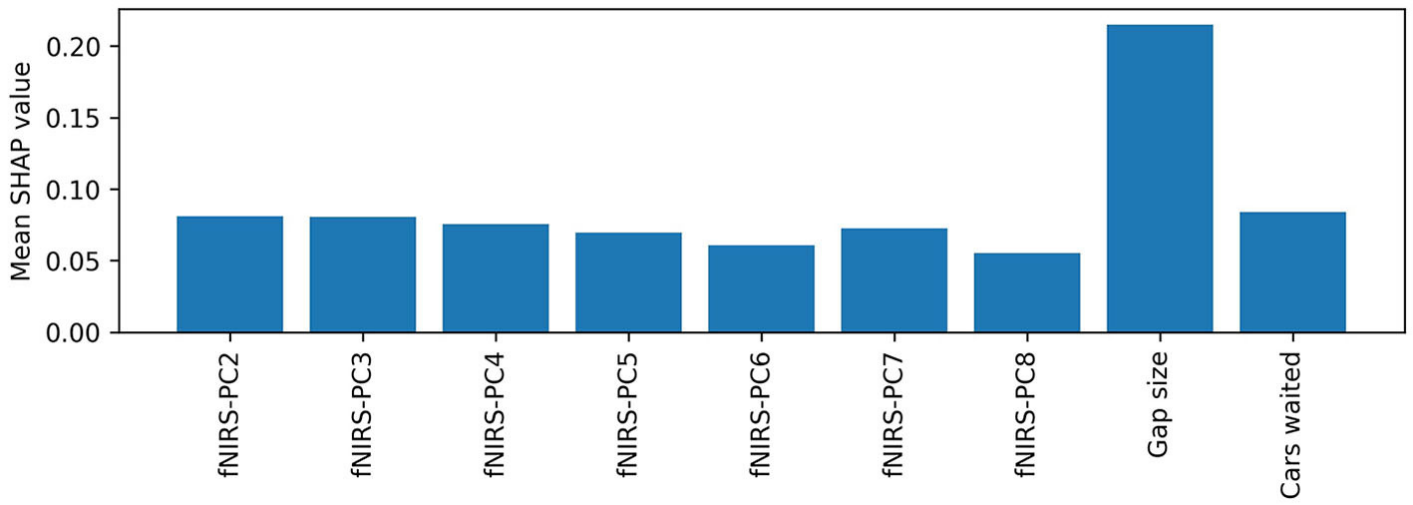
Mean absolute SHAP values for each feature of the combined model. The features are sorted by descending SHAP values.

Feature importance analysis can be used to evaluate the contribution of the model's input variables to the model's classification. We used SHAP to investigate which variables contribute most to the turning intention recognition. The mean SHAP value of each variable and thus the average impact on the model output is shown in Figure 7. It can be seen that “Gap Size” has the highest impact on the model outcome with a mean SHAP value of 0.21. It is followed by the principal components from the fNIRS test data. The second fNIRS principal component has a mean SHAP value of 0.11.

**Figure 8:**
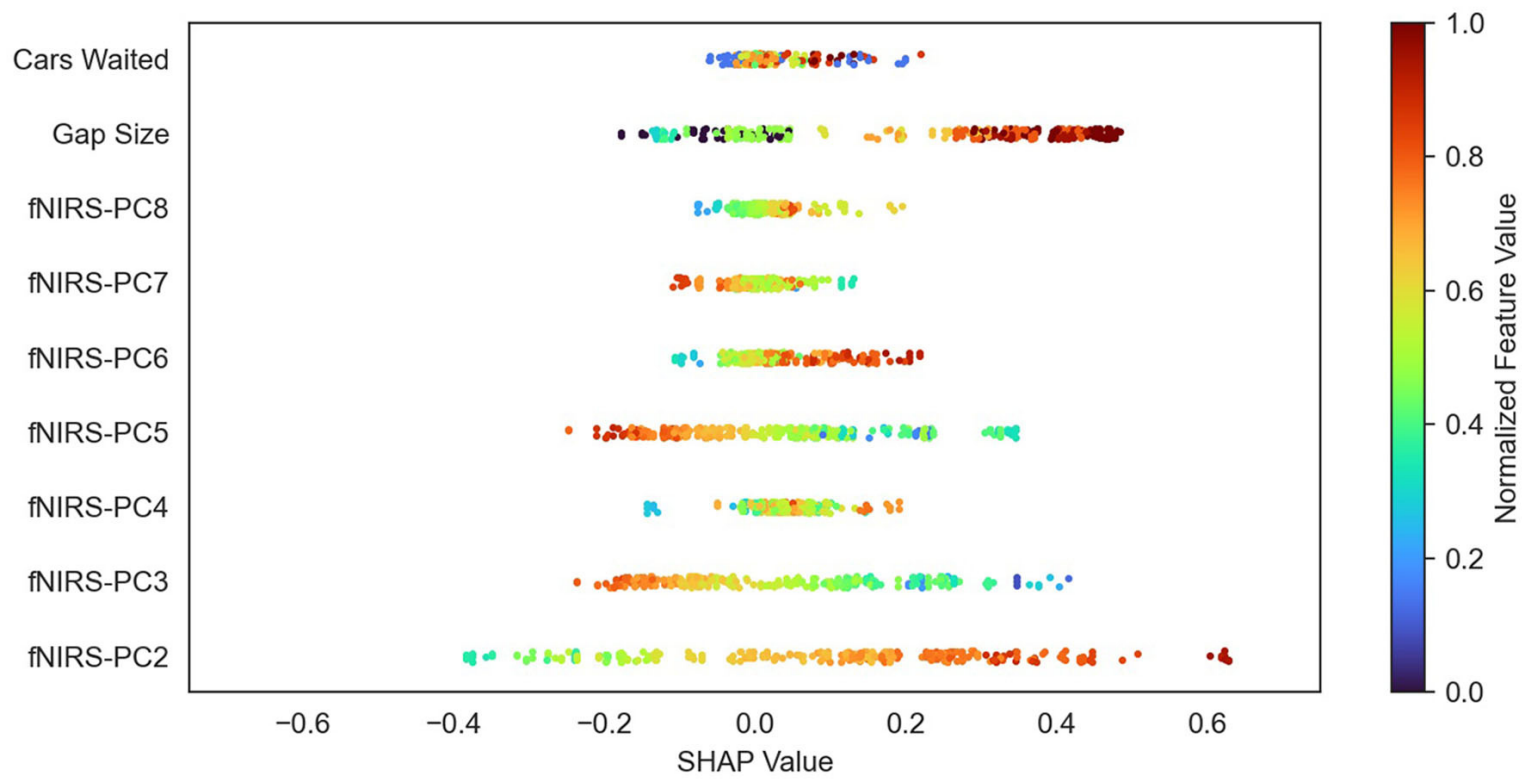
Beeswarm plot of the feature importance analysis of one subject. Each dot represents one sample of the test dataset. SHAP values for each of these samples are represented by their value on the *x*-axis. The normalized feature values of each sample are color-coded. Note how the feature values vary systematically in the features with high average SHAP values.

Figure 8 shows a beeswarm plot for the feature importance analysis for one subject and one test dataset of one iteration of the k-fold cross-validation. Each dot represents one sample of the test dataset used. The SHAP values for each of these samples are represented by their value on the *x*-axis. The feature value of each sample is color-coded and normalized for visualization purposes. One can see, based on the color coding, that wide gap sizes and number of cars (red) waited have higher positive SHAP values. This indicates that the model will most likely classify turning in instances, where such feature values are present.

### Effect size analysis and functional brain areas

**Figure 9:**
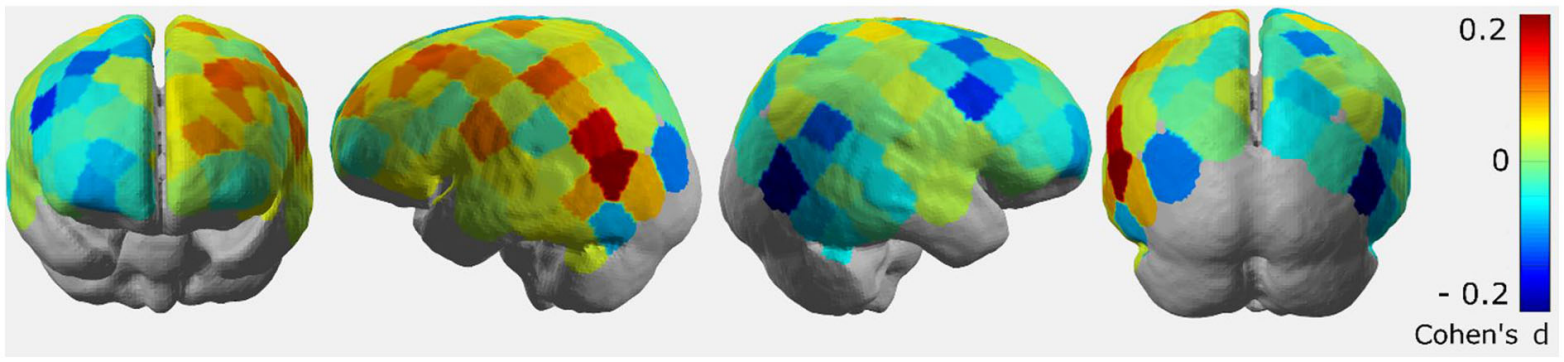
Group-level average Cohen's *d* brain maps depicting effect sizes computed from channel-wise averaged t-statistics. A tendency for lateralization of activation over the brain is visible.

To explore which brain areas may be predictive of the turning decision, we computed average Cohen's *d* brain maps from *t*-values computed for the difference between “turn” and “no turn” activation levels for each channel separately (Figure 9) We visualized the average Cohen's *d* brain map on the MNI 152 brain using MRIcron^2^ to determine MNI coordinates and the corresponding putative Brodmann areas (BA) with increased effect sizes. Table 1 lists the brain areas, the MNI coordinates of the difference maxima, and the average Cohen's *d* values. Note the small range of Cohen's *d* values (−0.2–0.2) in Figure 9 is due to group-level averaging. On a single-subject level, Cohen's *d* values range from −0.95 to 1.10.

**Table 1:** Brain areas showing differential activation effects in the decision-making phase during the intention to turn as compared to the waiting phase.

**Brain areas**	**Putative brodmann area (BA)**	**X**	**Y**	**Z**	**Cohen's d**
Left primary motor cortex	4	−56	−10	44	0.15
Left premotor area	6	−26	8	70	0.08
Left supplementary motor area	8	−24	20	62	0.12
Left middle frontal gyrus	9	−10	56	36	0.10
Left middle temporal gyrus	21	−64	−48	0	0.15
Left inferior parietal lobule	39	−54	−62	22	0.20
Right primary motor cortex	4	18	−30	78	0.05
Right premotor cortex	6	24	2	74	−0.15
Right middle frontal gyrus	9	48	16	50	−0.15
Right middle temporal gyrus	37	58	−64	14	−0.20

The approximate MNI coordinates of activation differences along with the putative Brodmann areas and their Cohen's d values are shown.

Our results showed the largest effect sizes for brain activation differences in the left inferior parietal lobule (putative BA 39) for “turn” compared to “no-turn” conditions (average Cohen's *d*~0.2). Additionally, parts of the motor cortices, such as the left primary motor cortex (PMC; putative BA 4), left supplementary motor area (SMA; putative BA 8), and left premotor area (putative BA 6), also indicate an increased activation difference (average Cohen's *d* ~ 0.1), for “turn” compared to the “no turn” condition. These areas have previously been implicated in action planning and movement execution (van der Kallen et al., 1996; Kapreli et al., 2006). Furthermore, some informative channels can be seen in the left middle frontal gyrus (putative BA 9) and the left middle temporal gyrus (putative BA 21).

## Discussion

Our aim was to develop a model for the recognition of driver turning intention using contextual experimental data and neurophysiological measures. For this, we performed an fNIRS-driving simulator study where participants had to wait at an unsignalized intersection and perform a left-turn maneuver through a stream of oncoming vehicles. The quantitative analysis of our DNN modeling approach showed that a model with both neurophysiological and contextual information is able to considerably better classify the intent of the driver to turn as compared to using only the contextual information or the neurophysiological information. Especially with respect to false negatives (no turn predicted when a turn was performed), the inclusion of neurophysiological measures provides an additional independent source of information for driver intent recognition, which may reduce the amount or criticality of these severely safety-critical situations. Our turning intention recognition model with just fNIRS data achieves a median accuracy of 83.1%, which is already higher than the accuracies reported in Lin et al. (2018). The context model has a similar accuracy of 83.8%, which is slightly lower than the 85% accuracy of the model presented by Phillips et al. (2017). Although the paradigm differs between our model and Phillips et al. (2017), incorporating more contextual information like intersection layout may help to improve our model's performance. The combined model achieved a median classification accuracy of 91.9%. This accuracy is above the results from Lin et al. (2018), who investigated a similar paradigm and used an electroencephalograph to classify turning maneuvers. The model proposed by Zhang and Fu (2020) achieves a slightly higher accuracy of 94.2% based on contextual and vehicle data, like the vehicle's position, speed, and acceleration. These prediction rates are higher than our context model's accuracy (83.8%) and the accuracy of our combined model (91.9%). Reasons for this may be that the researchers had access to more contextual variables, like the position or speed of the vehicle. Furthermore, a time window of 11 s was used for the prediction in comparison to our 4 s time window for classification.

The results of the ROC analysis presented in Figure 6 show that the context model has an average AUC of 0.92 ± 0.04 and the combined model has an AUC of 0.94 ± 0.02. Although the AUCs seem to be close to each other the combined model has a lower variability between subjects regarding the AUC. For some participants, the context model can achieve AUC values close to the combined model. However, for others, the AUC is considerably worse. We hypothesize that these participants have in some intersections shown turning behavior that cannot easily be modeled with a context-only model. Since the context-only model has just two input variables, namely the gap size and waiting time, the model will learn to recognize the driver's intention based on a combination of these two, which will most likely represent an acceptable gap size with respect to the waiting time or vice versa. The context model will not be able to make the correct classification if the participant will decide to use a smaller than usual gap size in some of the intersections. Using a smaller gap size than normally preferred could for example be caused by the time pressure applied during the experiment. In these situations, the combined model seems to be able to utilize information from the fNIRS brain activation measurements to help classify these uncommon turning decisions with a smaller than usual gap size, which leads to an overall more consistent performance of the model in comparison to the context only model.

**Figure 10:**
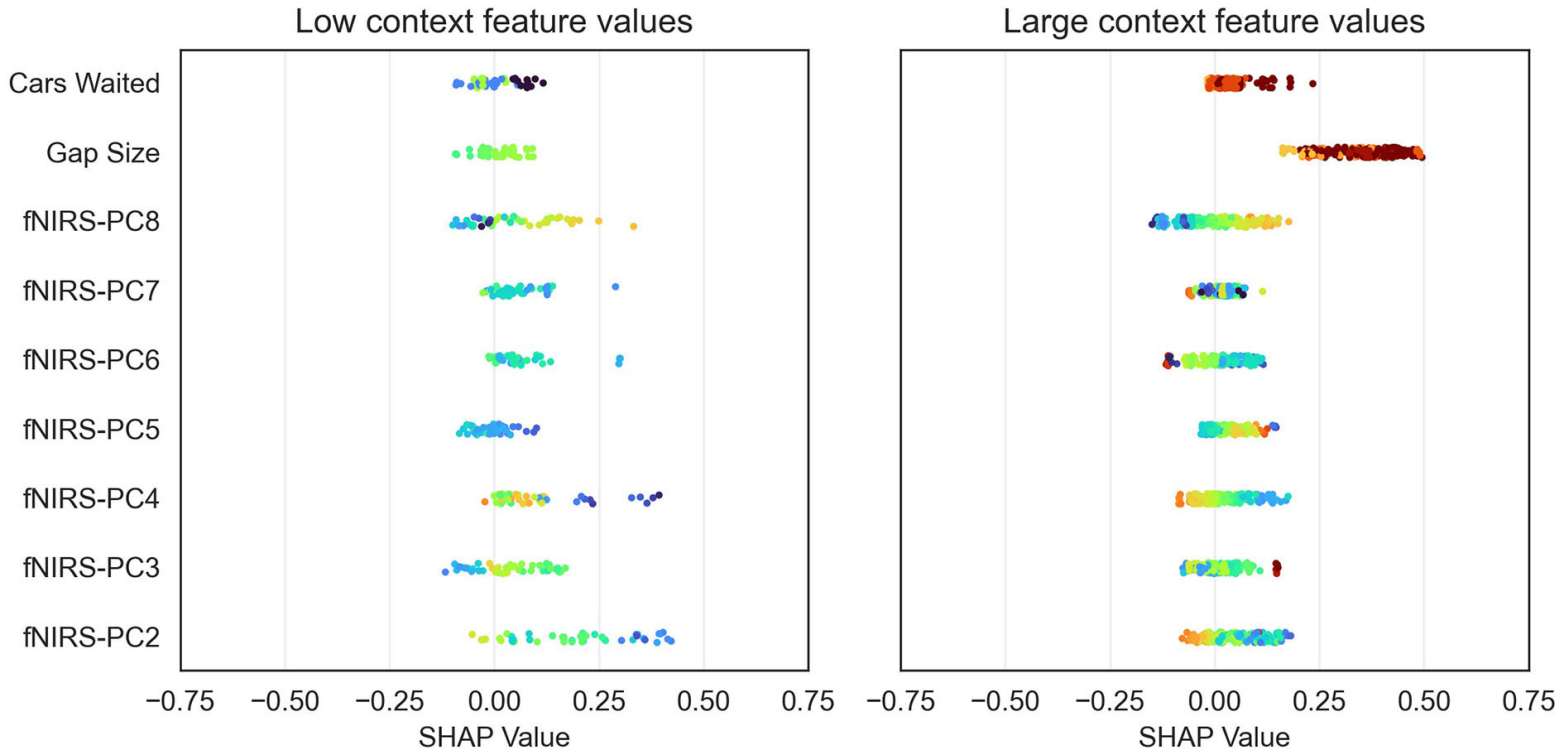
Beeswarm plots comparison of low and high context feature values for one subject and false negatives of the context only model. Left: Only samples with low context feature values. The combined model learned to utilize the brain activation features for the correct classification. This is indicated by the higher fNIRS SHAP values. Right: For large context feature values, the impact of the context features is on average higher than the brain activation information.

To better understand the contribution of contextual and fNIRS features for turning intent recognition, we performed a SHAP feature importance analysis. Our results suggest that both variables “waiting time” and “gap size” are important for the model output. However, the mean SHAP value for the gap size was nearly three times as high as the waiting time's SHAP value. These results are in line with results from previous gap-acceptance studies for left-turning situations suggesting that the gap size is the most important variable during the turning decision-making process (Ragland et al., 2005; Yan et al., 2007). Some researchers found that the waiting time also has an impact on the driver's turning intent (Fricker et al., 1991; Zohdy et al., 2010). The analysis also showed that most of the principal components (PCs) from the fNIRS data have an equal impact on the model's output than the waiting time. A more in-depth analysis of the SHAP values (Figure 8) showed that the model can utilize information in certain PCs for a “turn” and “no turn” classification, respectively. As expected, larger gap sizes and higher waiting times have larger positive SHAP values and thus contribute more to a “turn” classification. The combination of fNIRS and context information can reduce the number of safety-critical false negatives as indicated by the confusion matrices (Figure 5). A potential basis of this effect is suggested by looking separately at the data of misclassified trials with low or high context feature values in the combined model. Figure 10 shows two SHAP value beeswarm plots for the combined model of one subject. The left plot only contains samples that have below-average context feature values, meaning that these were instances with low gap sizes and cars waiting. Conversely, the right plot shows only samples with above-average context feature values. Furthermore, the figures show only samples where the context-only model would create false negatives, thus classifying “no turn” where “turn” would have been correct. All samples shown in the figures are correctly classified by the combined model. It can be seen that for the low context feature values (left), where the subject merged after short waiting times and into small gap sizes, the fNIRS features tend to have higher SHAP values than the context features. This indicates that they contribute most to the model's correct classification. For the large context feature values (i.e., longer waiting times and wider gaps), the gap size has the highest impact on the model output. Although both fNIRS and waiting time have an impact on the model's correct classification, they appear to contribute less to the classification than gap size. This suggests that the combined model can use information from the fNIRS features to make a correct classification in situations where a context-only model would fail to make the correct classification. In this example, the information from fNIRs seems to be most important in atypical turns with small gaps and short waiting times. This suggests that the brain data provide information independent of the context and supports the notion that this affects the subject's decision to turn.

One advantage of whole-head fNIRS in complex realistic tasks is that it allows for the analysis of distributed brain networks that contribute to the observed behavior (Unni et al., 2017). The analysis of the predictive fNIRS channels in the decision-making phase comparing the “turn” vs. “no turn” phases revealed brain activity predictive for turning in brain areas that are thought to underly a wide range of functions important for safe turning through oncoming traffic like visual processing, evidence accumulation, decision-making, executive control of behavior, and the execution of motor sequences. Among the areas listed in Table 1, the posterior part of the middle temporal gyrus (putative BA 21) has been shown to be involved in the observation of motion, such as tracking a stream of oncoming vehicles at the intersection (Kolster et al., 2010). The BA 39, potentially including the caudal part of the intraparietal sulcus has been shown to be involved in executive control of behavior, visuospatial processing, spatial focusing of attention, and executing a sequence of actions (Köhler et al., 1995; Crozier et al., 1999; Buchsbaum et al., 2006; Kübler et al., 2006). Putative BA 9 in the left middle frontal gyrus (putative BA 9) has been associated with executive functions, such as decision-making and action planning (Rogers et al., 1999; Fincham et al., 2002; Zhang et al., 2003; Babiloni et al., 2005). Moreover, activation modulations in left BA 4, BA 6, and BA 8 may indicate the preparation for the execution of the turning maneuver as they include brain areas important for the execution of complex movement sequences, such as the primary motor cortex (PMC; putative BA 4), premotor area (PMA; putative BA 6), and supplementary motor area (SMA; putative BA 8). While the primary motor cortex in BA 4 is implied in the control of muscle movements (Vogt and Vogt, 1919; van der Kallen et al., 1996; Kapreli et al., 2006), PMA and SMA have been associated with visuomotor coordination, such as preparation and planning of movement-execution and interlimb coordination (Freund, 1990; Stephan et al., 1995; Crozier et al., 1999; Ehrsson et al., 2000; Rämä et al., 2001; Schubotz and von Cramon, 2001).

The current study has a few limitations. First, the left-turning scenario has limited context variables. Besides gap size and waiting time, there are not many other context variables to include in the model. Previous studies on gap acceptance in such scenarios suggest demographic variables, such as gender, age, and driving style (Pollatschek et al., 2002; Yan et al., 2007; Trende et al., 2019), have an impact on the decision-making in these situations and may help to further improve a recognition model. However, this approach is not possible for the subject-wise modeling approach used in this study. Furthermore, the sample size of 13 participants is relatively small. Small sample sizes are common for studies involving neurophysiological measurements due to the complex experimental setup and data processing pipeline. However, the analyses were here performed at the single subject level to demonstrate the feasibility of turning prediction in a realistic driving simulator scenario. Due to the limited transfer of classification models across subjects, it is necessary to perform this type of analysis. Group-level studies in traffic research commonly involve dozens up to hundreds of participants or recorded vehicles, which lead to a more detailed picture of the drivers' behavior in a given situation. However, they typically investigate average behavior instead of attempting predictions of behavior in single situations, as it was done here, and as is relevant to the design of an actual safety function used *in situ* as a sub-function of vehicle control. Furthermore, we used only fNIRS HbR data for the modeling approach since the HbO signal by itself has been shown to be far more susceptible to systemic physiological artifacts compared to HbR. This results in the possibility of inadvertently measuring the hemodynamic responses that are not caused due to neurovascular coupling and misinterpreting them as brain activity (Tachtsidis and Scholkmann, 2016). Hence, these “false positives” in the fNIRS data are more likely to be observed in HbO than HbR. Additionally, fNIRS studies using connectivity metrics to better understand brain networks in complex decision-making tasks and user state estimation are gaining popularity (Senoussi et al., 2017; Dehais et al., 2018; Verdière et al., 2018). While the PCA approach that we used as input features in our model could be considered as one such type of network analysis, the interpretation of the PCs is often difficult. Future studies could explore other connectivity methodologies which can provide better insights into the brain dynamics while driving.

We hypothesize that a working intention recognition model could help to prevent critical situations or even accidents when integrated into a vehicle control function (Damm et al., 2019). An intention recognition model could warn the driver *via* a user interface or initiate an emergency braking maneuver. Including brain activation measures into such models may be particularly helpful in atypical turns, where the brain activation may provide valuable information independent of the context and reduce the number of misclassifications, particularly in risky situations. With the rise of Vehicle-to-Vehicle communication, such a system could also be used to warn the oncoming vehicle in time to slow down and thus increasing the gap size.

## Data availability statement

The raw data supporting the conclusions of this article will be made available by the authors, without undue reservation.

## Ethics statement

The studies involving human participants were reviewed and approved by Ethics Committee of the Carl von Ossietzky University, Oldenburg. The patients/participants provided their written informed consent to participate in this study.

## Author contributions

AT, AU, MJ, BB, MF, AL, and JR contributed to the conception and design of the study. AT and AU performed the study. AU and MJ performed the fNIRS analysis. AT created and validated the models and wrote the first draft of the manuscript. All authors contributed to manuscript revision, read, and approved the submitted version.

## Funding

This work was supported by the DFG-grants RI 1511/3-1 to JR, LU 1880/3-1 to AL, BE4532/15-1 to BB (all Learning from Humans—Building for Humans), and FR 2715/4-1 (Integrated Socio-technical Models for Conflict Resolution and Causal Reasoning) to MF.

## Conflict of interest

The authors declare that the research was conducted in the absence of any commercial or financial relationships that could be construed as a potential conflict of interest.

## Publisher's note

All claims expressed in this article are solely those of the authors and do not necessarily represent those of their affiliated organizations, or those of the publisher, the editors and the reviewers. Any product that may be evaluated in this article, or claim that may be made by its manufacturer, is not guaranteed or endorsed by the publisher.
